# Soluble AXL as a marker of disease progression and survival in melanoma

**DOI:** 10.1371/journal.pone.0227187

**Published:** 2020-01-09

**Authors:** Karine Flem-Karlsen, Marta Nyakas, Inger Nina Farstad, Erin McFadden, Patrik Wernhoff, Kari Dolven Jacobsen, Vivi Ann Flørenes, Gunhild Mari Mælandsmo

**Affiliations:** 1 Department of Pathology, The Norwegian Radium Hospital, Oslo University Hospital, Oslo, Norway; 2 Institute for Clinical Medicine, Faculty of Medicine, University of Oslo, Oslo, Norway; 3 Department of Tumor Biology, Institute for Cancer Research, The Norwegian Radium Hospital, Oslo University Hospital, Oslo, Norway; 4 Department of Oncology, The Norwegian Radium Hospital, Oslo University Hospital, Oslo, Norway; 5 Institute of Medical Biology, Faculty of Health Sciences, UiT–Arctic University of Norway, Tromsø, Norway; University of Queensland Diamantina Institute, AUSTRALIA

## Abstract

Receptor tyrosine kinase AXL is a one-pass transmembrane protein upregulated in cancers and associated with lower survival and therapy resistance. AXL can be cleaved by the A Disintegrin and Metalloproteinases (ADAM)10 and ADAM17, yielding a soluble version of the protein. Elevated soluble AXL (sAXL) has been reported to be associated with disease progression in hepatocellular carcinoma, renal cancer, neurofibromatosis type 1 and inflammatory diseases. In the present work, we analyzed sAXL levels in blood from melanoma patients and showed that sAXL increases with disease progression. Additionally, increased sAXL levels were found correlated with shorter two-year survival in stage IV patients treated with ipilimumab. Furthermore, we showed that sAXL levels were related to the percentage of cells expressing AXL in resected melanoma lymph node metastases. This finding was verified *in vitro*, where sAXL levels in the cell media corresponded to AXL expression in the cells. AXL inhibition using the small-molecular inhibitor BGB324 reduced sAXL levels, while the cellular expression was elevated through increased protein stability. Our findings signify that quantification of sAXL blood levels is a simple and easily assessable method to determine cellular AXL levels and should be further evaluated for its use as a biomarker of disease progression and treatment response.

## Introduction

Melanoma is among the cancers with the highest increase in incidence worldwide [1]. Treatment of melanoma is challenging due to high intratumoral heterogeneity and therapy resistance [2–4]. Currently, immunotherapies, such as monoclonal antibodies targeting CTLA-4 and PD-1, have become first line treatment. While the response is quite favorable in a fraction of the patients, these treatments are costly and come with significant side effects and there is currently no method for identifying non-responding patients [5]. Additionally, small-molecular inhibitors targeting the ERK/MAPK pathway, which comprise BRAF and MEK inhibition may be suitable for patients with BRAF mutated tumors. However, many patients become resistant, leading to disease progression [6]. Thus, there is a need for biomarkers to select therapy and monitor treatment resistance and disease recurrence in melanoma patients.

Receptor tyrosine kinase AXL constitutes the TAM family together with TYRO3 and MER [7]. The TAM family members share GAS6 as their ligand, although AXL has the highest binding affinity. Upon ligand binding, the receptor dimerizes and autophosphorylates, leading to activation of downstream signaling pathways such as PI3K and ERK/MAPK [8]. Induced expression of AXL is observed in several cancer forms and correlates with disease progression and decreased survival [9, 10]. Additionally, AXL has been implicated in treatment resistance to immunotherapy, targeted therapies and chemotherapy, where higher expression of AXL is observed in resistant compared to sensitive cells [11–15].

AXL is a one-pass transmembrane protein, with its extracellular portion consisting of two immunoglobulin-like domains and two fibronectin type III domains [16]. Proteolytic cleavage of the N-terminal domain of AXL by the metallo-endopeptidases A Disintegrin and Metalloproteinases (ADAM)10 and ADAM17 [17], sheds a ~80–85 kda extracellular fraction [17, 18] known as soluble AXL (sAXL). sAXL is present in human serum and has been demonstrated to be elevated in hepatocellular [19] and renal cell carcinoma [20]. Further, we recently reported that sAXL levels were higher in patient effusions from ovarian carcinoma, malignant mesothelioma and breast cancer compared to benign reactive effusions [21]. Additionally, we found that sAXL levels were elevated in effusions from high-grade versus low-grade serous ovarian carcinomas [21]. On the other hand, an engineered AXL decoy receptor consisting of the extracellular domain has been found to act as a decoy by binding GAS6, resulting in decreased metastatic potential [22].

We aimed to investigate the feasibility of using sAXL in serum and plasma as a biomarker for disease progression in metastatic melanoma and examine if sAXL levels could be related to tumor burden. We showed that the level of sAXL mirrors the levels of cellular AXL in melanoma cell lines and blood samples, and that treatment with the AXL inhibitor BGB324 or ERK/MAPK inhibitors reduced the levels of sAXL in cell media. Of particular interest, increased sAXL levels in blood samples from melanoma patients were correlated with disease progression. Furthermore, we found that elevated sAXL levels in stage IV melanoma patients treated for seven weeks with ipilimumab significantly correlated with disease progression and reduced survival.

Together, our data suggest that sAXL blood levels may be exploited as an easily assessable marker to monitor cellular AXL expression and that increased levels of sAXL in late-stage patients should be further evaluated as a marker of treatment failure and disease progression.

## Results

### sAXL is present in media from melanoma cell lines and the levels are reduced by AXL or MAPK inhibition

**Fig 1:**
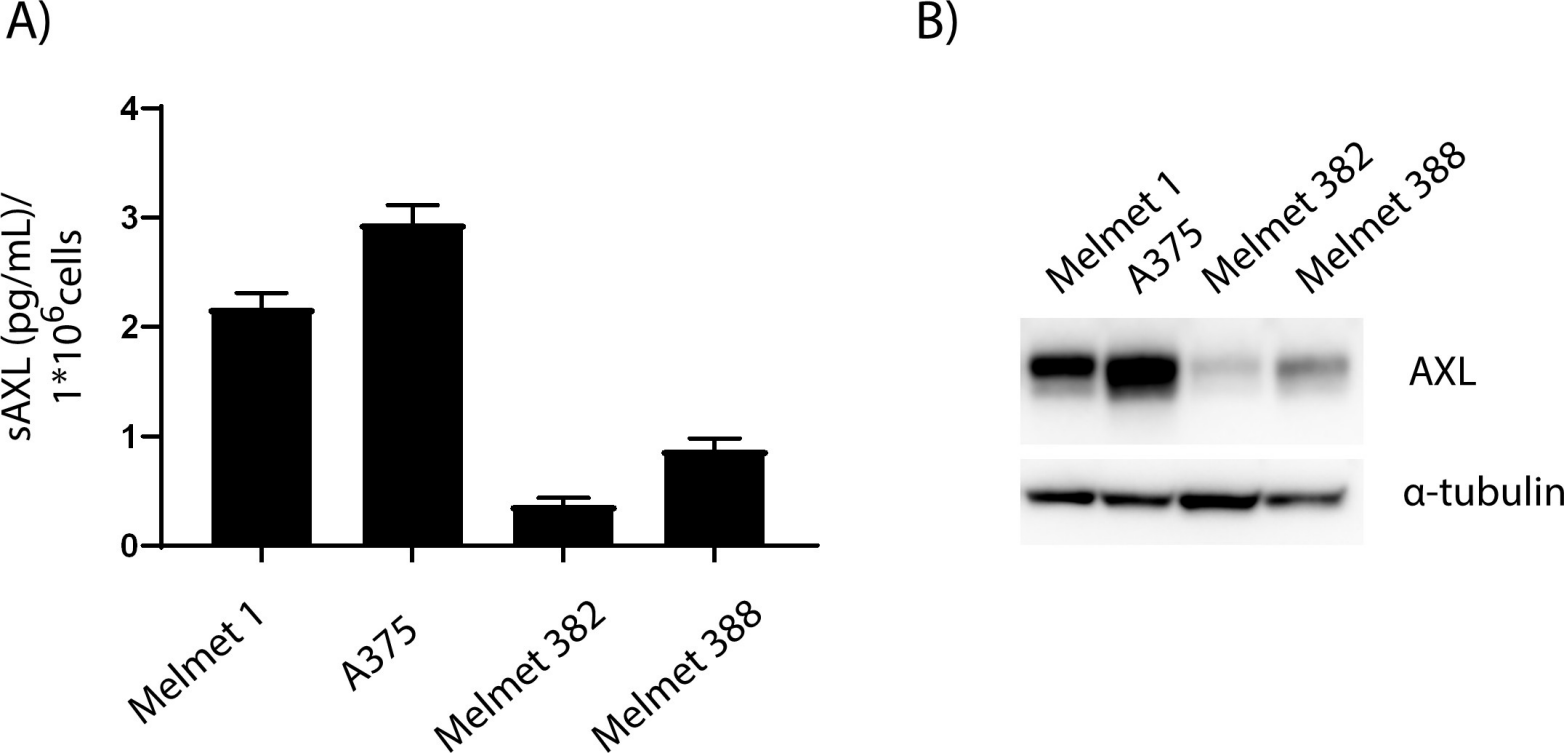
sAXL mirrors AXL levels in cell lines. A) Levels of sAXL as measured by ELISA in the media of melanoma cell lines Melmet 1, A375, Melmet 382 and Melmet 388 related to 1*10^6^ cells. Error bars indicate SEM (n = 3) and B) Corresponding cellular AXL levels as measured by immunoblot. α-tubulin was used as loading control.

We observed sAXL in the media of four melanoma cell lines with AXL protein expression (Fig 1A and 1B), while no sAXL was detected in the AXL-negative cell line Melmet 369 (S1A and S1B Fig). The cellular protein expression of AXL reflected the amount of sAXL in the media of the respective cell line. To determine if the level of sAXL detected was expressed as a soluble isoform or contained within extracellular vesicles, we deprived media of extracellular vesicles by ultracentrifugation before measuring sAXL levels (S1C Fig). Following removal of extracellular vesicles, the levels of sAXL were reduced by ~20% indicating that the soluble isoform is the main contributor to sAXL.

**Fig 2:**
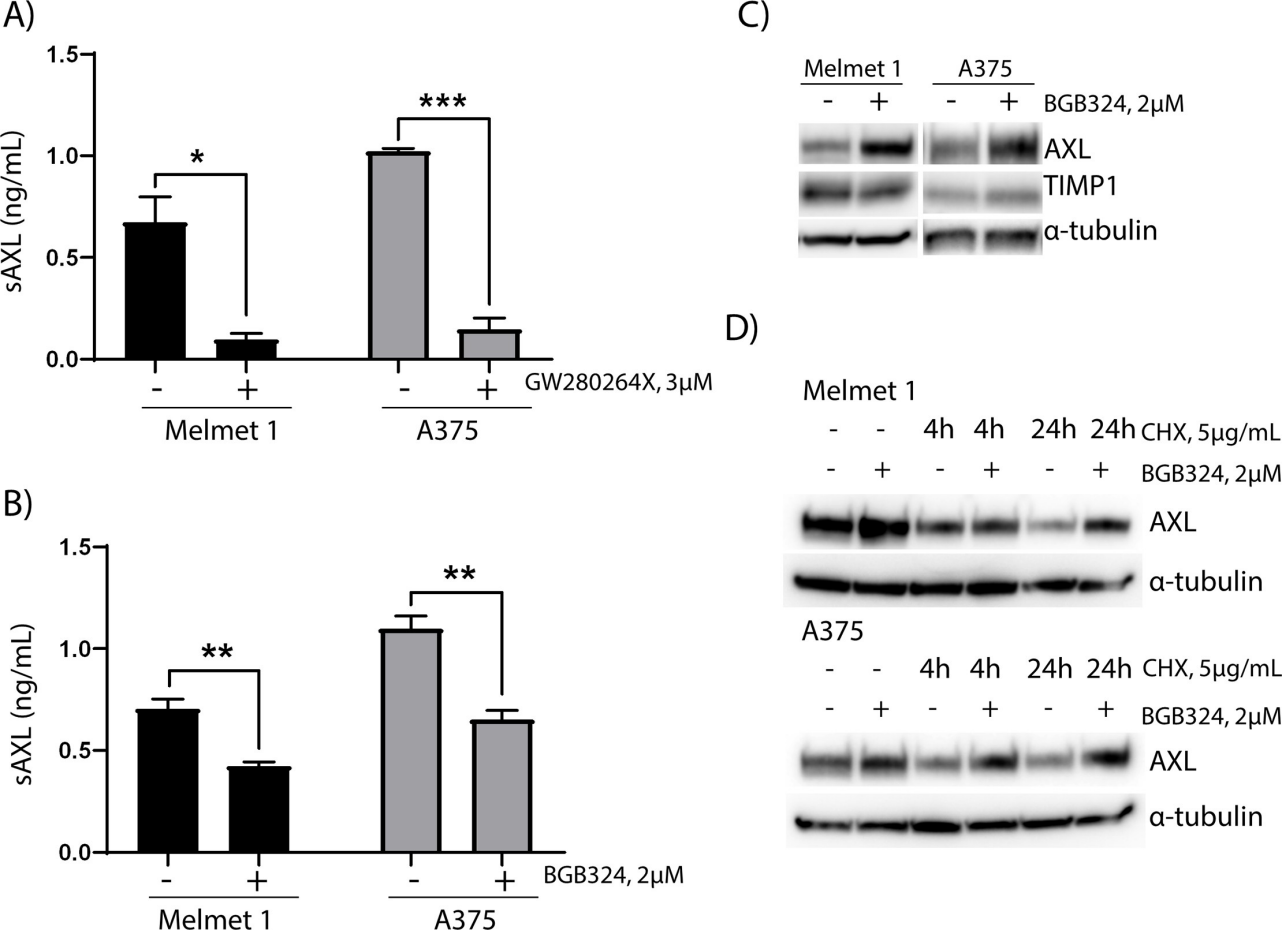
AXL inhibition results in reduced sAXL levels and augmented AXL expression through increased protein stability. sAXL levels in the media of Melmet 1 and A375 cells treated with A) 3 μM ADAM10/ADAM17 inhibitor GW280264X and B) 2 μM BGB324. sAXL levels were determined by ELISA. For B, the control cells are the same as in Fig 3A and 3B. The figures show average sAXL levels + SEM of three independent experiments. Immunoblot analyses showing protein expression of C) AXL and TIMP1 following treatment with 2 μM BGB324 and D) AXL following treatment with 2μM BGB324 and/or 5 μg/mL protein synthesis inhibitor cycloheximide (CHX). Cells were treated with BGB324 for 24 hours and/or CHX for indicated times before harvesting. α-tubulin was used as loading control. * = p value ≤ 0.05, ** = p value ≤ 0.01 and *** = p value ≤ 0.001 calculated using student two-tailed t-test.

Recently, AXL has been found to be cleaved by ADAM10 and ADAM17 to yield sAXL [17]. In support of this, treatment with GW280264X, an inhibitor of ADAM10 and ADAM17, abolished the levels of sAXL in Melmet 1 and A375 cell media (Fig 2A) (p value = 0.0104 and 0.0001, respectively), and increased AXL cellular levels, without affecting cell proliferation (S2A and S2B Fig). To determine if reduced AXL activity altered the amount of sAXL, we treated Melmet 1 and A375 cells with BGB324, a small-molecular inhibitor targeting AXL [23]. The results showed increased cellular expression of AXL, while sAXL levels in the media were reduced by 30–40% following BGB324 treatment (Fig 2B and 2C) (p value Melmet 1 = 0.006 and A375 = 0.004), without affecting proliferation (S2C Fig) The increased cellular expression of AXL was not caused by increased transcription, as no increase in AXL mRNA levels following treatment with BGB324 was observed (S2D Fig). It has been reported that BGB324 induces mRNA expression of the ADAM inhibitor TIMP1 [24]. Although this implies that BGB324 may reduce ADAM10/17 activity and thereby cause less cleavage of AXL and increased membrane expression of the protein, we did not observe increased TIMP1 protein levels upon BGB324 treatment in our cell lines (Fig 2C). Furthermore, it was recently proposed that treatment with an AXL inhibitor could increase the protein stability [25]. Accordingly, we observed elevated AXL expression in cells treated with BGB324 and the protein synthesis inhibitor cyclohexemide compared to cyclohexemide alone (Fig 2D). These data showed that inhibiting AXL activity results in increased protein stability and reduced cleavage of the protein.

**Fig 3:**
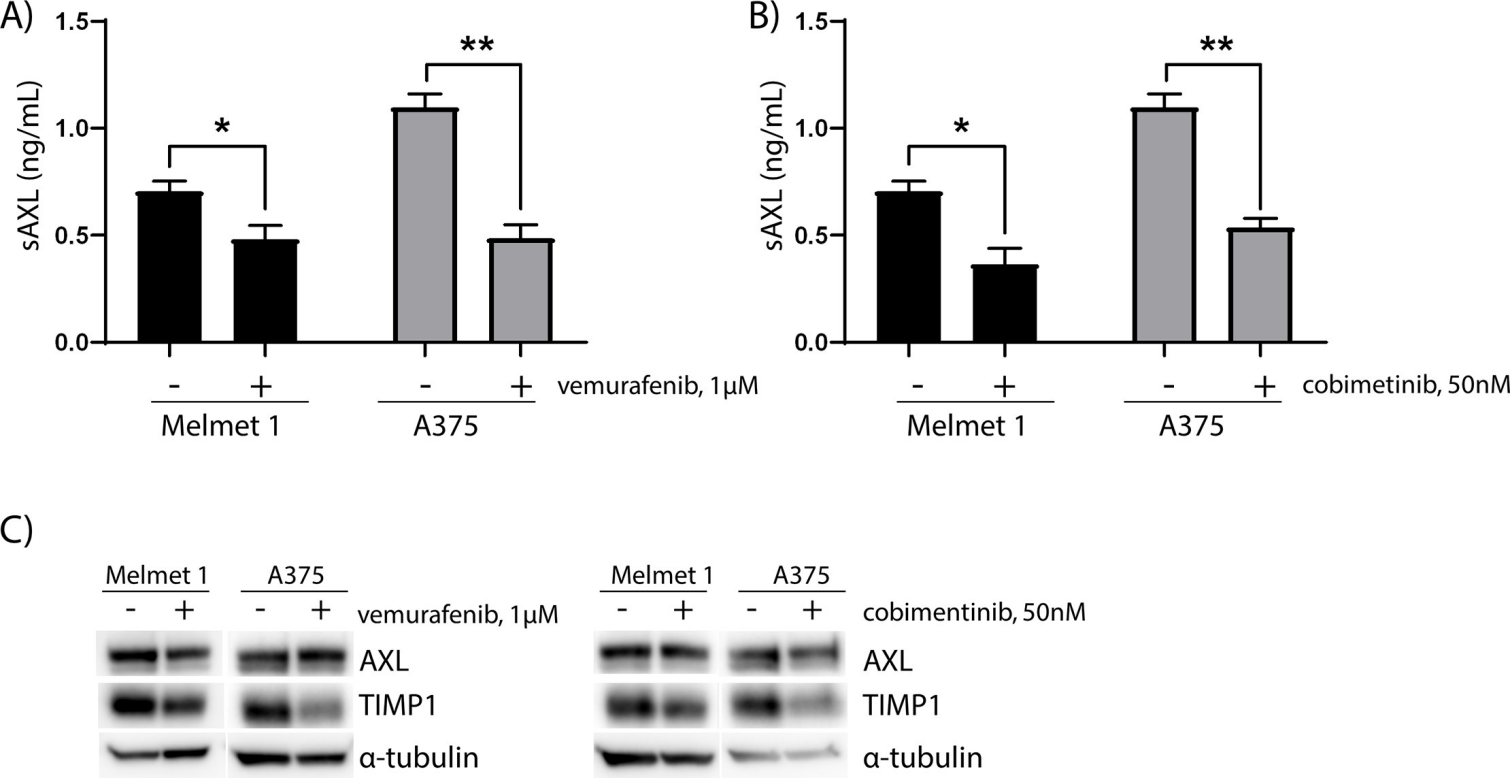
MAPKi results in reduced sAXL without affecting AXL expression. sAXL levels in the media of Melmet 1 and A375 cells treated with A) 1μM vemurafenib and B) 50nM cobimetinib and C) AXL and TIMP1 cellular levels in the corresponding cell lines as visualized by representative immunoblots. Cells were treated with the inhibitors for 24 hours before media or cells were harvested. sAXL levels were determined by ELISA and show average sAXL levels + SEM of three independent experiments. For A and B, the control cells are the same as in Fig 2B. α-tubulin was used as loading control for the immunoblot. * = p value ≤ 0.05 and ** = p value ≤ 0.01 calculated using student two-tailed t-test.

Reduced sAXL levels were also observed in the media of cells treated with the ERK/MAPK inhibitors vemurafenib or cobimetinib (Fig 3A and 3B) (p value Melmet 1 = 0.05 and 0.02 and A375 = 0.002 and 0.002, respectively), without having an effect on cell proliferation (S2C Fig). Interestingly, TIMP1 expression was reduced in vemurafenib and cobimetinib treated cells, while AXL cellular levels were unchanged (Fig 3C). This suggests that the reduced sAXL levels in vemurafenib and cobimetinib treated cells are not effectuated by increased TIMP1 expression, in contrast to previous reports [17]. When treating cells with BGB324, vemurafenib and cobimetinib in combinations, no change in sAXL levels were observed between mono- and combination therapies (S3 Fig).

### sAXL levels increase with disease progression and confers with cellular AXL protein expression

**Fig 4:**
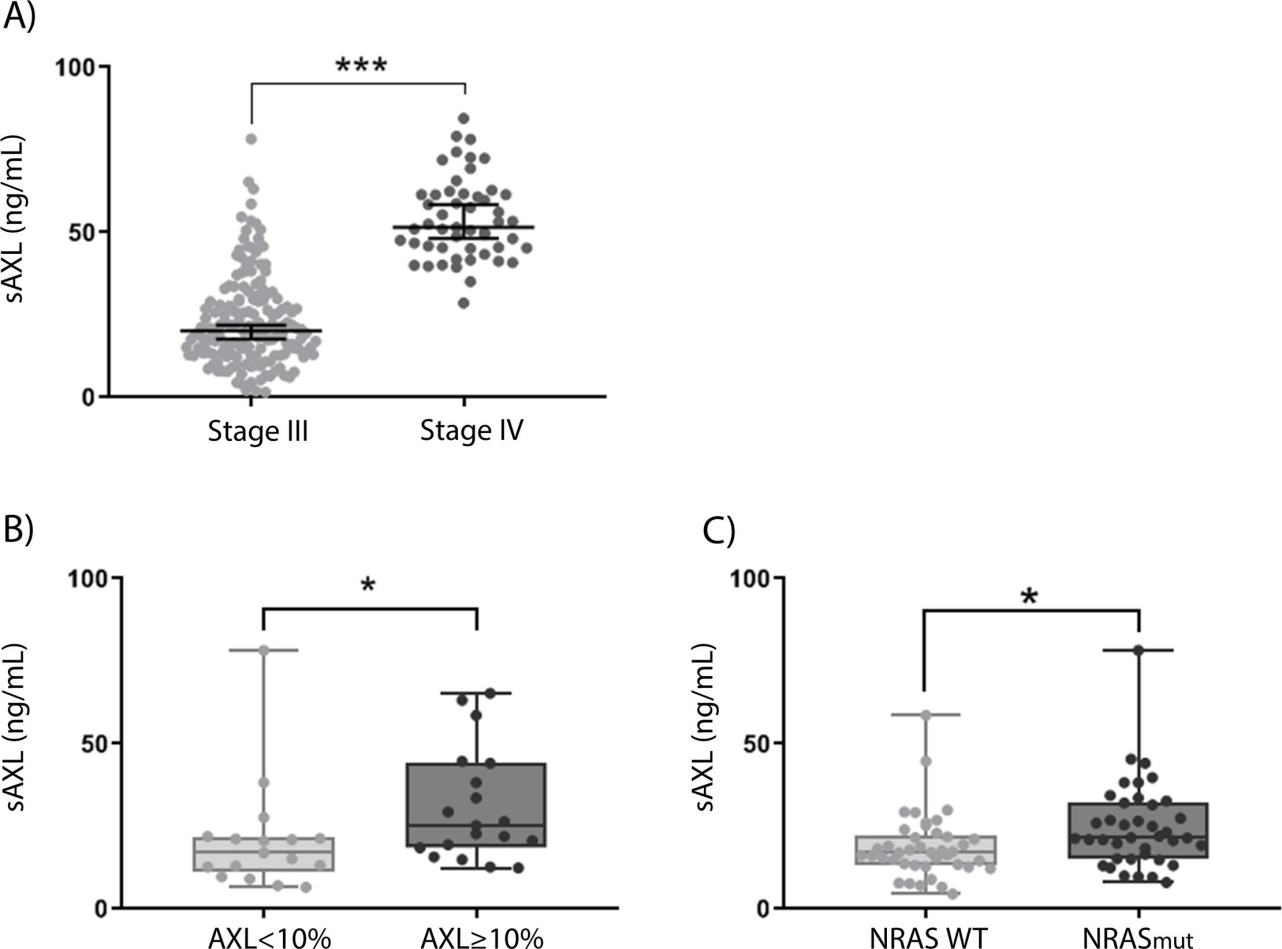
sAXL levels increases during melanoma progression and correlates with cellular AXL levels. A) sAXL levels measured by ELISA in blood harvested from patients either at time of lymph node resection (stage III) or at the start of ipilimumab treatment (stage IV) (n = 160 and 50, respectively). Error bars represent mean ± 95% confidence interval (CI). B) sAXL levels in 36 plasma samples related to immunohistochemical (IHC) staining of AXL in the respective lymph node metastases. Percent IHC staining expression was divided into two similar sized groups of <10% and ≥10% AXL staining (n = 17 and 19, respectively). C) sAXL levels in plasma drawn from stage III patients at lymph node resection related to NRAS mutation status. B) and C) are displayed as box and whiskers plot ± range. sAXL levels were determined by ELISA from plasma samples run in technical duplicates, where each point represents one patient. * = p value ≤ 0.05 and *** = p value ≤ 0.001.

To examine if sAXL could be detected in blood from melanoma patients, we collected blood samples from patients at the time of lymph node resection (stage III disease) or at the start of ipilimumab treatment (stage IV disease) (n = 160 and 50, respectively). sAXL was detected in all samples analyzed, with a range of 7.9 to 84.5 ng/mL. Patient characteristics are detailed in Tables 1 and 2. As seen in Fig 4A, mean sAXL expression increased from 26.6 ng/mL (95% CI = 24.3–28.9 ng/mL) in patients at lymph node resection to 54.1 ng/mL (95% CI = 50.7–57.6 ng/mL) in patients at the start of ipilimumab treatment (p value < 0.0001), with an area under the curve of 0.9256 (S4A Fig). To evaluate whether there were differences in TIMP1 levels between stage III and IV melanoma patients, we analyzed publically available TCGA data and found no change in TIMP1 mRNA levels (S4B Fig). The level of sAXL in stage III patients was not associated with overall survival, Breslow tumor thickness, ulceration, age or gender (S4C Fig and S1 Table). To examine if the level of sAXL coincide with the protein expression of AXL in tumor cells, paraffin embedded sections from 36 lymph node metastases were stained with an AXL antibody (S5 Fig). The immunohistochemistry scores were compared with the respective sAXL levels in the patient blood samples drawn at the same time as lymph node surgery. Of these, 6 of the 36 lymph node metastases showed no AXL staining, while 21, 25 and 11 displayed staining in the membrane, cytoplasm and the nucleus, respectively. The staining localizations were combined and patients with tumors expressing high levels of AXL (expression in ≥10% cells) had a corresponding higher plasma level of sAXL (Fig 4B) (p value = 0.0231). Additionally, we observed higher sAXL levels in patients with NRAS mutation compared to NRAS wild type (Fig 4C) (p value = 0.0143), conferring with a previous report showing higher AXL cellular levels in NRAS mutated melanoma cell lines [26]. The data indicate that the levels of sAXL mirror the expression of cellular AXL.

**Table 1:** Patient characteristics of the study population stage III disease.

	N (%)*
**N**	160
**Gender**	
Male	104 (65.0)
Female	56 (35.0)
**Age, median (range)**	65 (25–94)
**Melanoma subtype**	
Superficial spreading	43 (26.9)
Nodular	42 (26.3)
Other (acral, desmoplastic)	8 (5.0)
Unknown	67 (41.9)
**Breslow, mm (range)**	2.5 (0.4–25)
**Ulceration**	41 (49.4)**
**Mitotic index, pr mm2 (range)**	4 (0–37)
**BRAF V600 mutation**	
Yes	77 (48.1)
No	76 (47.5)
Unknown	7 (4.4)

*Data are shown as number of patients (%) unless otherwise indicated.

** Percentage of known data (n = 83).

**Table 2:** Clinical characteristics of the study population stage IV disease according to survival.

	Survival 2 years (%)	
Total N = 53^#^	Alive	Dead
** **	22 (41.5)	31 (58.5)
**Gender**		
Male	11 (20.8)	21 (39.6)
Female	11 (20.8)	10 (18.9)
**Age, median (range)**	60 (27–83)	67 (38–84)
**M stage**		
M1a/b	9 (17.0)	4 (7.5)
M1c	13 (24.5)	27 (50.9)*
**Organs involved**		
≤2	15 (28.3)	12 (22.6)
>2	7 (13.2)	19 (35.8)
**BRAF V600 mutation**		
Yes	12 (22.6)	13 (24.5)
No	7 (13.2)	16 (30.2)
Unknown	2 (3.8)	3 (5.7)
**LDH levels**		
Normal	19 (35.8)	13 (24.5)
>ULN†	3 (5.7)	18 (34.0)**
**Number of other treatments before inclusion**		
0	10 (18.9)	17 (32.1)
1	9 (17.0)	12 (22.6)
2	2 (3.8)	1 (1.9)
3	1 (1.9)	1 (1.9)
**Cardiovascular disease**		
No	20 (38)	27 (51)
Yes	2 (3,5)	4 (7.5)

#Number of patients at baseline = 50, week 4 = 50 and week 7 = 48. Total number of patients accumulates to 53 due to lacking blood samples of six patients at all three time points.

Data are shown as number of patients (%) unless otherwise indicated

†ULN upper limit of normal

*p<0.05

**p<0.01 *vs*. survivors.

### sAXL levels correlate with survival in stage IV melanoma patients treated with ipilimumab

**Fig 5:**
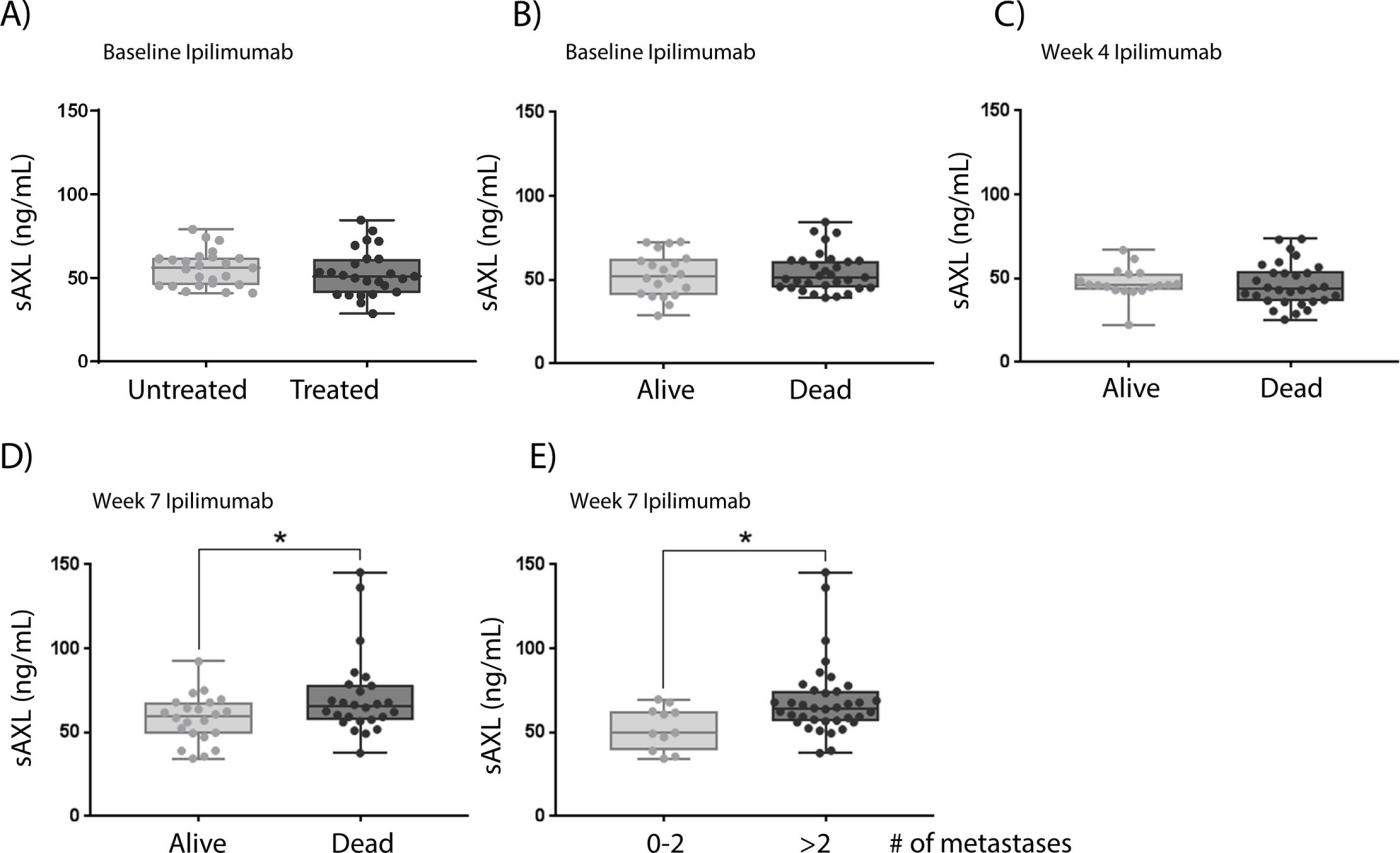
sAXL levels are increased in patients with shorter two-year survival after seven weeks of ipilimumab treatment. **A)** sAXL levels measured by ELISA in plasma samples in previously treated versus untreated patients at the start of ipilimumab treatment. sAXL levels in serum samples from stage IV patient harvested B) before the start, C) at week 4 or D) at week 7 of ipilimumab treatment grouped according to survival after two years. E) sAXL levels in serum harvested at week 7 of ipilimumab treatment related to number of metastases. sAXL levels were determined by ELISA and run in technical duplicates. The figures are displayed as box and whiskers plot ± range, where each point represents one patient. * = p value ≤ 0.05.

We further analyzed the level of sAXL in serum from patients who underwent ipilimumab treatment. Blood from a total of 53 patients was harvested at three time points; before the start of treatment (baseline), at 4 weeks (second course) and at 7 weeks of treatment (third course) (Table 2). Of the patients who started ipilimumab treatment, 25 of 50 had received previous therapy, where dacarbazine was the most prevalent. AXL has been linked to treatment resistance [27, 28], with higher AXL levels in chemotherapy resistant cells [29]. Therefore, we analyzed whether there was a change in the baseline sAXL levels in previously untreated versus treated patients. However, no difference was observed between these groups (Fig 5A).

Further, we aimed to examine if sAXL levels were associated with survival in stage IV melanoma patients treated with ipilimumab. There was no change in the expression of sAXL in serum drawn at baseline and week 4 when comparing patient survival two years after start of treatment (Fig 5B and 5C). Interestingly, in serum drawn from patients at week 7 of ipilimumab treatment (Fig 5D and S6 Fig), sAXL levels were increased in the patients who died within two years (71 ng/mL, 95% CI = 61.4–81.2 ng/mL) compared to those who were still alive (58.1 ng/mL, 95% CI = 51.8 ng/mL-64.4 ng/mL) (p value = 0.03). Higher sAXL levels were additionally observed in patients with more than two (69.0 ng/mL, 95% CI = 61.7–76.3 ng/mL) compared to patients with 0–2 metastases (52.6 ng/mL, 95% CI = 44.0–61.1 ng/mL) (p value = 0.023) (Fig 5E).

In this study, we found that sAXL levels mirror the levels of cellular AXL in melanoma cell lines and patient samples. Further, we showed that sAXL levels increase with disease progression, and that stage IV patients who had higher levels of sAXL at week 7 of ipilimumab treatmenthad shorter two-year survival. Together, these data demonstrated the potential of measuring sAXL in blood as a non-invasive method to monitor cellular AXL levels and showed that sAXL may be used to predict disease progression in melanoma patients.

## Discussion

In the present study, we observed that inhibition of AXL by the small-molecular inhibitor BGB324 resulted in reduced levels of sAXL, and increased the expression of the cellular protein. However, increased expression of AXL was not observed in cells treated with vemurafenib or cobimetinib, despite displaying decreased levels of sAXL. It has been previously reported that the MEK inhibitor PD325901 reduces the catalytic activity of ADAM10 and ADAM17, through increased mRNA expression of TIMP1 [17]. Likewise, BGB324 in combination with an EGFR inhibitor has been shown to increase the mRNA expression of TIMP1 in glioblastoma cells [24]. In contrast to this, we observed reduced TIMP1 expression in ERK/MAPK inhibited cells. Furthermore, we observed no increase in TIMP1 protein expression after BGB324 treatment, suggesting cell specific mechanisms to be responsible for the AXL cleavage in these cells. For instance, inhibition of ubiquitination and increased protein stability has been reported associated with AXL cell surface accumulation in response to AXL inhibition in breast and lung cancer cells [25]. This is in accordance with our results, demonstrating increased AXL expression in cells treated with BGB324 in combination with the protein synthesis inhibitor cycloheximide.

We have previously observed that although the protein expression of AXL is increased in BGB324-treated cells, the activity is decreased compared to control cells (Flem-Karlsen, *in revision*). Thus, the membrane-bound, but kinase inactive, AXL may decrease downstream signaling by binding and sequestering its ligand GAS6. Further, the extracellular domain of AXL may activate the receptor present on other cells ligand-independently [30]. Hence, treatment with AXL inhibitors may prevent excessive proliferation not only through downregulation of their respective pathways, but also by decreased activation of AXL on other cells.

The addition of an engineered extracellular domain of AXL has been shown to reduce disease progression and therapy resistance by acting as a decoy of GAS6 [22, 31]. However, these studies have generated libraries of AXL mutants with a high affinity for GAS6 to act as an inhibitor of the GAS:AXL pathway. Although sAXL is found to bind GAS6 in serum and plasma, only a fraction of sAXL was bound to GAS6, indicating a surplus of AXL [32]. This suggest that excess sAXL may activate membrane-bound AXL through AXL homodimerization. Furthermore, GAS6 activation of AXL has been suggested to play a less dominant role in settings where AXL is overexpressed [33], such as in cancer.

In line with a previous publication in hepatocellular carcinoma [34], we observed a positive correlation between the expression of cellular AXL and the level of sAXL in media from melanoma cell lines. These data indicate that measuring sAXL could be exploited to determine the amount of cellular AXL expressed in the tumor cells. No correlation between sAXL in serum and the mRNA expression in the respective tumor was reported in renal cancer [20]. It has additionally been reported that AXL mRNA expression was similar in dendritic cell and macrophages, despite one having abundant, while the other had minimal AXL protein expression [35]. This suggests a tight post-transcriptional regulation of the protein, highlighting the necessity to relate sAXL levels to the protein expression of AXL.

AXL expression has been linked to metastasis, treatment resistance and poor survival [8, 28] [36], thus monitoring the levels of AXL in patients may be a tool to determine if the patient tumors display aggressive tumor characteristics. We observed higher sAXL levels in melanoma patients at stage IV compared to stage III. Our observed result is in concordance with others, showing higher sAXL levels in later-stage hepatocellular and renal cancers [19, 20]. Currently, disease relapse is monitored through CT scans, which expose the patients to radiation. sAXL levels predicted melanoma stage with good sensitivity and specificity and could be evaluated as a biomarker of disease progression which may reduce the need for CT scans. sAXL was detected in serum and plasma from stage III and stage IV patients, respectively, meaning that the comparison between these two groups must be done with caution. However, sAXL expression in serum versus plasma from Alzheimer patients has been reported to yield significantly similar levels [37]. Additionally, sAXL has been reported to show consistency across a range of parameters, such as different storage conditions, buffer types, freeze/thaw cycles and dilutions, highlighting the stability of this protein in blood [38].

There are currently no approved biomarkers of immunotherapy response in melanoma. In this study, we showed that the level of sAXL in serum samples measured after 7 weeks of treatment (third course) with ipilimumab was higher in patients who died within two years. Additionally, we showed that the levels of sAXL mirror the levels of cellular AXL. Measuring sAXL levels could thus be a measure of the amount of tumor cells that display the treatment resistant AXL^high^ phenotype, highlighting the potential to distinguish patients that have increased stage and less response to treatment. Additionally, AXL is reported to be involved in signaling which leads to immune suppression [39]. Thus, the higher sAXL levels in patients with poor prognosis suggest that the patients had lower immune activation and that single-agent immune therapy might not be sufficient. In line with this, AXL inhibition in combination with PD-1 inhibitor pembrolizumab is currently in phase Ib/II clinical trial for treatment of metastatic melanoma (NCT02872259). Our data suggest that measuring sAXL levels may be an easy method to identify patients that need more aggressive treatment regimens and/or closer follow-up. Importantly, sAXL levels may be evaluated from routine blood samples and may be measured over time to monitor alterations in AXL expression. However, it may be difficult to determine treatment response on sAXL levels alone, due to somewhat overlap between the two groups in ipilimumab treated patients at seven weeks. To increase specificity and sensitivity, sAXL should be further studied in a panel together with other markers that are associated with cancer aggressiveness.

In stage III patients, no correlation was observed between sAXL levels and overall survival. In these patients, the levels of sAXL released from the tumor cells may be too low compared to the overall sAXL expression released from normal cells to be able to distinguish patients based on survival. Thus, measuring sAXL levels in combination with other markers may prove more beneficial to increase the assay sensitivity for this group of patients.

In conclusion, we observed higher sAXL levels in late-stage melanoma patients compared to patients at an earlier stage, and sAXL levels were linked to a higher number of metastases and lower survival at week 7 of treatment. Furthermore, we observed a correlation between cellular AXL expression and sAXL levels in melanoma cell lines and patient samples, suggesting that measuring sAXL may be used as an easy assessable marker to determine disease progression and aggressiveness. Thus, monitoring disease progression of both Stage III and IV melanoma patients may reduce the number of required CT scans and thereby, the amount of radiation for each patient over time.

## Materials and methods

### Patient material and cell lines

Blood samples were obtained from melanoma patients treated at the Norwegian Radium Hospital, Oslo University Hospital. Samples were either drawn at the time of lymph node metastasis resections, for patients with stage III disease, or before- and at week 4 (at second course) and week 7 (at third course) for stage IV patients treated with 3 mg/kg ipilimumab. Ipilimumab was given every third week up to four courses. Patients with inflammatory diseases were excluded from receiving ipilimumab. Peripheral venous blood was drawn into Vacuette® Na-Citrate 3,2% tubes (Med-Kjemi AS, Asker, Norway) for plasma samples and Vacuette® Serum Gel tubes (Med-Kjemi AS, Asker, Norway) for serum samples. After coagulation at room temperature, tubes were centrifuged at 2,500 g for 20 minutes for plasma and 1,500 g for 10 minutes for serum, and the samples were stored at -80°C in multiple aliquots. Metastatic lymph node melanoma specimens were obtained from stage III melanoma patients who underwent surgery at the Department of Plastic and Reconstructive Surgery, The Norwegian Radium Hospital, Oslo University Hospital between 1990 and 2016. The histologic diagnosis was based on World Health Organization criteria, and the pathologic staging was performed according to the tumor, node and metastatic classification system AJCC7. Patient material was collected in accordance with the Declaration of Helsinki with informed consent and was approved by the Norway Regional Committee for Medical and Health Research Ethics (application numbers 2014/2208, 2015/2434 and 2013/1518). NRAS and BRAF^V600E/K^ mutations were determined by routine diagnostics by an in-house PCR based assay. Melanoma cell lines Melmet 1, Melmet 369, Melmet 382 and Melmet 388 were established from metastatic lesions of patients treated at the Norwegian Radium Hospital, Oslo University Hospital. A375 was obtained from American Type Culture Collection (Manassas, VA, USA). Melmet 1, A375 and Melmet 382 cells are BRAF^V600E^ mutated, while Melmet 369 and Melmet 388 cells are NRAS^Q61^ mutated. Extensive sequencing data of the tumors Melmet 369, Melmet 382 and Melmet 388 cell lines were generated from are available at Flørenes *et al*, Transl Oncol, 2019 [40]. The cell lines were routinely checked for mycoplasma, and Melmet 1 and A375 were STR fingerprinted. Cells were grown in RPMI-1640 (Sigma Aldrich, St. Louis, MO, USA) supplemented with 5% fetal bovine serum (FBS, Sigma Aldrich, St. Louis, MO, USA) and 2 mM L-glutamine (Lonza Bioscience, Basel, Switzerland) and kept at 37°C and 5% CO_2_.

### Immunoblot and protein analysis

Protein lysates were lysed in a buffer comprising of 1% Triton X-100, 50mM Hepes (pH 7.4), 150mM NaCl, 1.5Mm MgCl_2_, 1mM EGTA, 100mM NaF, 10mM Na Pyruvate, 1mM Na_3_VO_4_ and 10% Glycerol, with addition of 10 μL/mL protease and phosphatase inhibitor cocktails (cOmplete Mini and PhosSTOP^™^, Roche, Mannheim, Germany). Protein quantification was determined by Bradford analysis (Bio-Rad Laboratories AB, Sundbyberg, Sweden). 25μg protein/lane was run on SDS polyacrylamide gel electrophoresis (PAGE) before the protein was transferred to a PDVF immobilon membrane (Millipore, Bedford, MA). Membranes were blocked with 5% non-fat milk in 0.1% TBS-T (150 mM NaCl, 25 mM Tris-Cl, (pH 7.5), 0.01% Tween 20), before incubation with primary antibodies overnight at gentle agitation. Antibodies used were: AXL (#8661) and TIMP1 (D19E6, #8946) (1:1000, Cell Signaling, Boston, MA, USA), and α-tubulin (DM1A) (#05–829, 1:50.000, Millipore, Burlington, MA, USA). The following day, membranes were washed 3x10 minutes in 0.1% TBS-T, hybridized with secondary antibody (HRP-conjugated anti-rabbit or anti-mouse (Promega)) with gentle agitation for one hour at room temperature before 3x10 minutes washes in 0.1% TBS-T. Protein bands were visualized by SuperSignal^™^ West Dura Extended Duration Substrate (Thermo Fisher Scientific, Waltham, MA, USA) and exposed in a Syngene G Box.

### Reagents

BGB324 was a kind gift from BerGenBio (Bergen, Norway). Vemurafenib and cobimetinib were purchased from Selleck Chemicals (Huston, TX, USA) and GW280264X was purchased from Aeobious Inc. (Gloucester, MA, USA). Cycloheximide solution (#C4859) was purchased from Sigma Aldrich (St. Louis, MO, USA). The inhibitors were diluted in DMSO and used at concentrations and time periods as indicated. Control groups received the same amount of DMSO as treated groups.

### Cell confluence

To determine the number of cells per well, cells were plated at 15–25% confluency in 96-well or 6-well plates and left overnight before treatment with drugs (or DMSO for control cells) and 400 ng/mL GAS6 and 10 μg/mL Vitamin K for 24 hours. Percent cell confluency was determined by IncuCyte FLR or IncuCyte Zoom Kinetic Imaging System (Essen Biosciences, Ann Arbor, MI, USA).

### Immunohistochemistry staining

To determine the protein expression of AXL in the melanoma lymph node metastases standard method immunohistochemistry was performed. Formalin-fixed, paraffin-embedded tissue specimens were deparaffinised with xylene and rehydrated in graded ethanol. Antigen retrieval was performed by boiling for 20 minutes at 97°C in Target Retrieval Solution buffer (pH 6,0: Dako, Glostrup, Denmark) in microwave oven. After quenching endogenous peroxidase with 3% H_2_O_2_ in methanol for 30 minutes, the slides where incubated over night at 4° with polyclonal antibody against AXL (#AF154, R&D Systems, Minneapolis, MN, USA) and labelled with the Envision Detection System (Dako, Glostrup, Denmark) for 1 hour at room temperature. The slides where developed with 3,3`-ddiaminobenzidine tetrahydrochloride (DAB Plus; Dako, Glostrup, Denmark) and counterstained with 10% Mayer hematoxylin, dehydrated, and mounted. AXL staining was evaluated by a pathologist (INF) blinded to patient characteristics. As no commonly accepted scoring system for *in situ* AXL expression is available, this was done semi-quantitatively with a subjective grading system for the proportion of tumor cells showing a positive reaction. In general, the pattern of AXL expression varied significantly among samples; the AXL protein could be located mostly to the cell membrane, in the cytoplasm or in nuclei, and in some samples, all expression patterns were present. The percentage of tumor cells showing membrane, cytoplasmic and/or nuclear staining was combined and recorded as <10%, 10–40% and over 50% for each sample. For analysis, AXL expression were divided into high (≥10%) and low (<10%), which generated two equally sized groups, in line with a previous publication [41].

### Enzyme-linked immunosorbent assay (ELISA)

The level of soluble AXL was quantified using Human Axl DuoSet® ELISA (Cat no. DY154, R&D Systems, Minneapolis, MN, USA) according to the manufacturer’s protocol. Plasma or serum samples were diluted 1:50 in reagent diluents, while cell media was undiluted. Media was removed of cells and apoptotic vesicles by centrifugation at 2000 g for 10 minutes before freezing. Cell culture supernatants abolished of extracellular vesicles were centrifuged at 100.000 g for 70 minutes by a Type Ti70 rotor (Beckman Coulter, Brea, CA, USA).

Samples were related to a sample standard of two-fold dilutions from 4000 pg/mL to 62.5 pg/mL and were measured in technical duplicates. Soluble AXL concentrations in Fig 1A were related to cell numbers counted manually by hematocytometer and presented as concentration (pg/mL)/10^6^ cells to account for variations in cell numbers. The other ELISA experiments are presented as concentration (ng/mL). The ELISA samples were normalized and quantitated using a second order polynomial standard curve by GraphPad Prism version 7.0 (GraphPad Software, San Diego, CA, USA).

### Statistical analysis

Values of cell-based experiments represent average of three independent experiments + standard error of the mean (SEM), if not otherwise noted. Statistical significance was determined by student two-tailed t-test using GraphPad Prism version 7.0 (GraphPad Software, San Diego, CA, USA). P-values of less than 0.05 were considered significant and marked with asterisks, where * = ≤ 0.05, ** = ≤ 0.01 and *** ≤ 0.001. Immunoblots were performed at least twice with independent lysates. Statistical analysis of patient samples was performed applying the SPSS-PC package (Version 25) using the Mann-Whitney U test (2-tier analyses) or the Kruskal Wallis H test (>2-tier analyses). Overall survival (; OS) was calculated from the date of diagnosis to recurrence or death, respectively. Univariate survival analyses of OS were executed using the Kaplan-Meier log-rank test. sAXL levels were classified as high vs. low based on the median value.

## Supporting information

S1 Fig sAXL levels mirror AXL cellular levels and is not primarily contained within extracellular vesicles. **A)** sAXL levels in the media of Melmet 369, Melmet 382 and Melmet 388 +SEM, and **B)** the corresponding AXL protein expression measured by immunoblot (N = 2). GAPDH was used as loading control. **C)** AXL levels in the media of Melmet 1 and A375 cells with or without depletion of extracellular vesicles by ultracentrifugation + SEM (n = 3). sAXL levels were measured by ELISA.
(TIF)

S2 Fig Treatments does not alter proliferation or mRNA expression in melanoma cells. A) Proliferation measured by Incucyte and **B)** Representative immunoblot of AXL protein expression of Melmet 1 and A375 cells treated with 3μM ADAM10/17 inhibitor GW280264X. α-tubulin was used as loading control for the immunoblot. B) Proliferation in Melmet 1 (top panel) and A375 (bottom panel) cells treated with 2 μM BGB324, 1 μM vemurafenib or 50 nM cobimetinib. Proliferation is measured by the Incucyte imaging system. C) Relative mRNA levels of AXL in Melmet 1 and A375 cells treated with 2μM AXL inhibitor BGB324. The data shows average values related to untreated control cells + SEM of three independent experiments. Cells were treated with the inhibitors for 24 hours before they were harvested.
(TIF)

S3 Fig Combinations of BGB324, vemurafenib and cobimetinib does not alter sAXL expression compared to monotherapies. sAXL levels in Melmet 1 (left panel) and A375 (right panel) cells treated with 2 μM BGB324, 1 μM vemurafenib and/or 50 nM cobimetinib for 24 hours. Control cells and monotreatment of BGB324, vemurafenib and cobimetinib are the same as the ones presented in Figs 2B, 3A and 3B. sAXL levels were determined by ELISA and show average values + SEM of three independent experiments.
(TIF)

S4 Fig sAXL levels increase with disease progression. **A)** Area under the curve (AUC) comparison between the levels of sAXL in patients at the start of ipilimumab treatment and at the time of lymph node resection. **B)** TIMP1 mRNA expression in stage III and IV melanomas from publically available TCGA data. **C)** Kaplan Meier plot of sAXL levels in blood divided in sAXL low (n = 80 and high (n = 80) from patients with stage III melanoma correlated with overall survival.
(TIF)

S5 Fig Immunohistochemistry staining of AXL. IHC staining showing examples of <10%, 10–50% and >50% AXL positive tumor cells in sections from stage III melanoma patients. <10%: Some cells in the lymph node metastasis (middle and right part of the picture) show faint cytoplasmic or nuclear staining. Stronger staining is seen in endothelial cells of lymphatic vessels (orig. magnif. X200). 10–50%: More cells in the metastasis (left part) show stronger staining, mainly cytoplasmic (orig. magnif. x100). >50%: More than half the cells in the metastatic node show relatively strong cytoplasmic and membrane staining (orig. magnify. X100).
(TIF)

S6 Fig sAXL levels are increased in patients with shorter two-year survival. AUC comparison between the levels of sAXL in patients who were alive or dead two years after ipilimumab treatment.
(TIF)

S1 Table Patient parameters grouped by median sAXL value. Ulceration, gender, age and Breslow depth sorted by high (n = 80) or low (n = 80) sAXL levels. Groups were determined by the median sAXL value.
(PDF)

S1 Supplementary Methods Methodological description of data mining related to S4B Fig. (DOCX)

S1 Raw Images Raw immunoblot images of blots used in the figures. Areas used in figures are indicated with blue brackets.
(PDF)
