# Supplementary figures and images for: Soluble AXL as a marker of disease progression and survival in melanoma
Source: PLoS One. 2020 Jan 9;15(1):e0227187. doi: 10.1371/journal.pone.0227187 (PMC6952099; doi:10.1371/journal.pone.0227187)

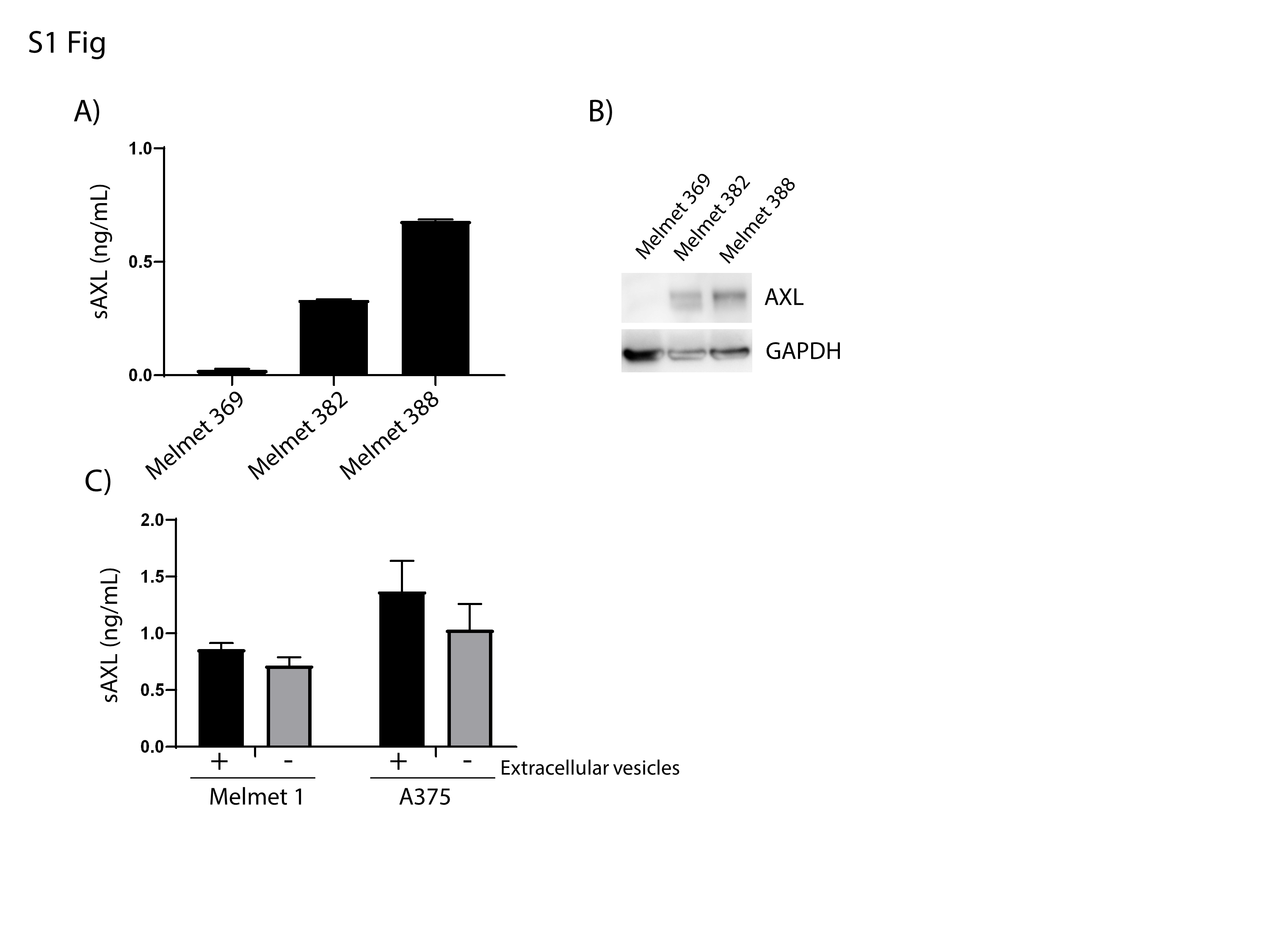

Supplement: S1 Fig — A) sAXL levels in the media of Melmet 369, Melmet 382 and Melmet 388 +SEM, and B) the corresponding AXL protein expression measured by immunoblot (N = 2). GAPDH was used as loading control. C) AXL levels in the media of Melmet 1 and A375 cells with or without depletion of extracellular vesicles by ultracentrifugation + SEM (n = 3). sAXL levels were measured by ELISA. (TIF) [file pone.0227187.s001.tif]

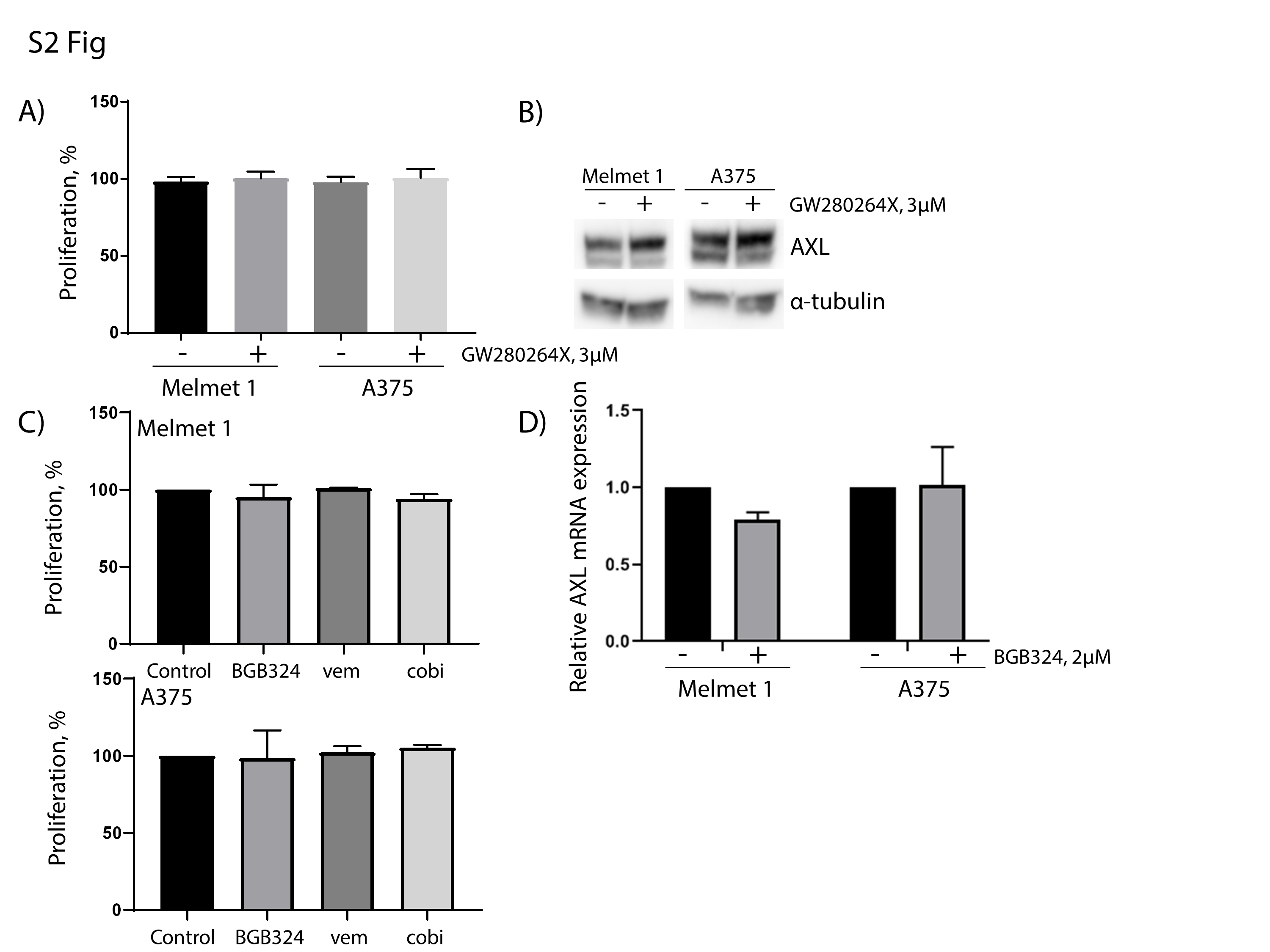

Supplement: S2 Fig — A) Proliferation measured by Incucyte and B) Representative immunoblot of AXL protein expression of Melmet 1 and A375 cells treated with 3μM ADAM10/17 inhibitor GW280264X. α-tubulin was used as loading control for the immunoblot. B) Proliferation in Melmet 1 (top panel) and A375 (bottom panel) cells treated with 2 μM BGB324, 1 μM vemurafenib or 50 nM cobimetinib. Proliferation is measured by the Incucyte imaging system. C) Relative mRNA levels of AXL in Melmet 1 and A375 cells treated with 2μM AXL inhibitor BGB324. The data shows average values related to untreated control cells + SEM of three independent experiments. Cells were treated with the inhibitors for 24 hours before they were harvested. (TIF) [file pone.0227187.s002.tif]

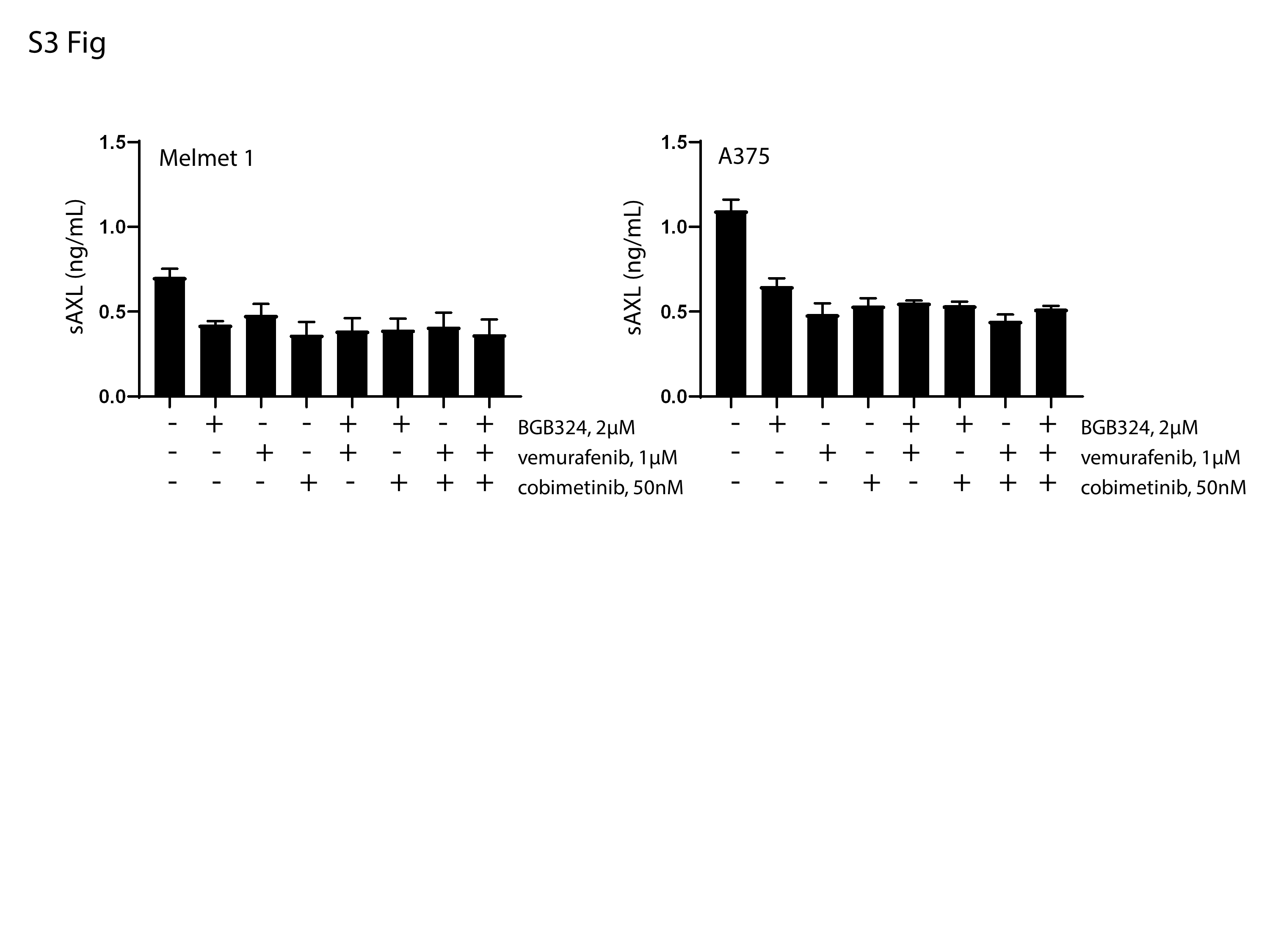

Supplement: S3 Fig — sAXL levels in Melmet 1 (left panel) and A375 (right panel) cells treated with 2 μM BGB324, 1 μM vemurafenib and/or 50 nM cobimetinib for 24 hours. Control cells and monotreatment of BGB324, vemurafenib and cobimetinib are the same as the ones presented in Figs 2B, 3A and 3B. sAXL levels were determined by ELISA and show average values + SEM of three independent experiments. (TIF) [file pone.0227187.s003.tif]

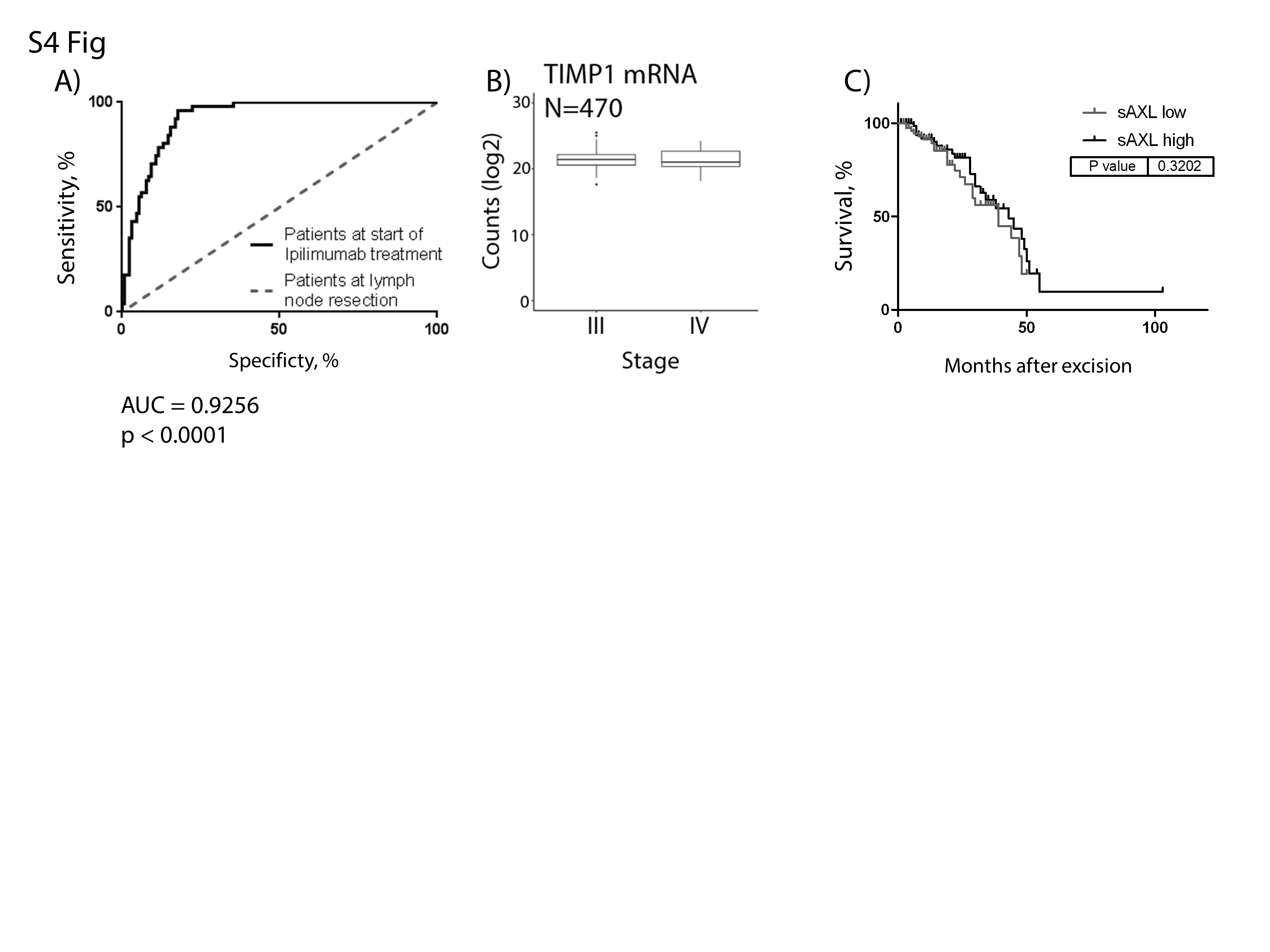

Supplement: S4 Fig — A) Area under the curve (AUC) comparison between the levels of sAXL in patients at the start of ipilimumab treatment and at the time of lymph node resection. B) TIMP1 mRNA expression in stage III and IV melanomas from publically available TCGA data. C) Kaplan Meier plot of sAXL levels in blood divided in sAXL low (n = 80 and high (n = 80) from patients with stage III melanoma correlated with overall survival. (TIF) [file pone.0227187.s004.tif]

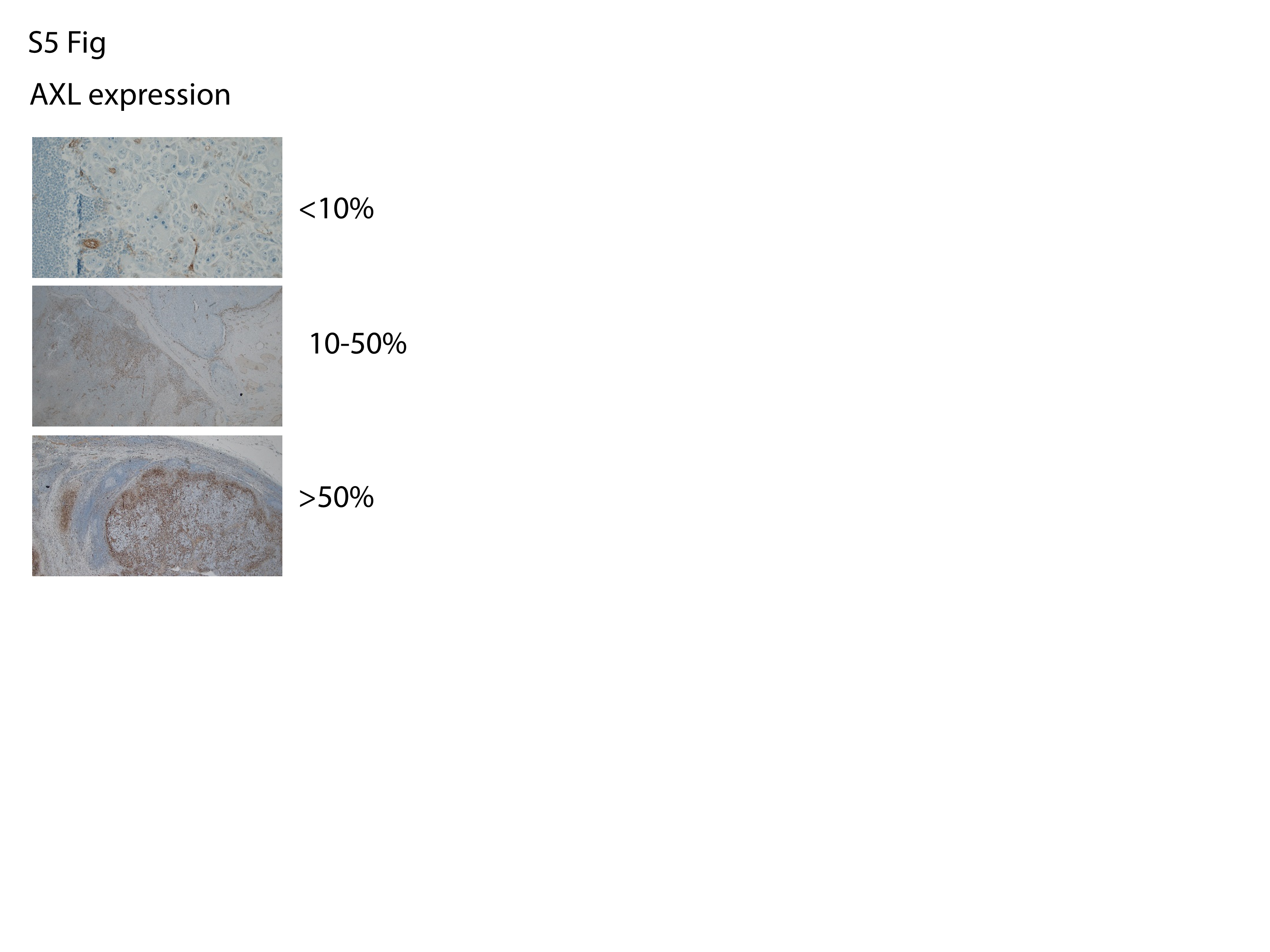

Supplement: S5 Fig — IHC staining showing examples of <10%, 10–50% and >50% AXL positive tumor cells in sections from stage III melanoma patients. <10%: Some cells in the lymph node metastasis (middle and right part of the picture) show faint cytoplasmic or nuclear staining. Stronger staining is seen in endothelial cells of lymphatic vessels (orig. magnif. X200). 10–50%: More cells in the metastasis (left part) show stronger staining, mainly cytoplasmic (orig. magnif. x100). >50%: More than half the cells in the metastatic node show relatively strong cytoplasmic and membrane staining (orig. magnify. X100). (TIF) [file pone.0227187.s005.tif]

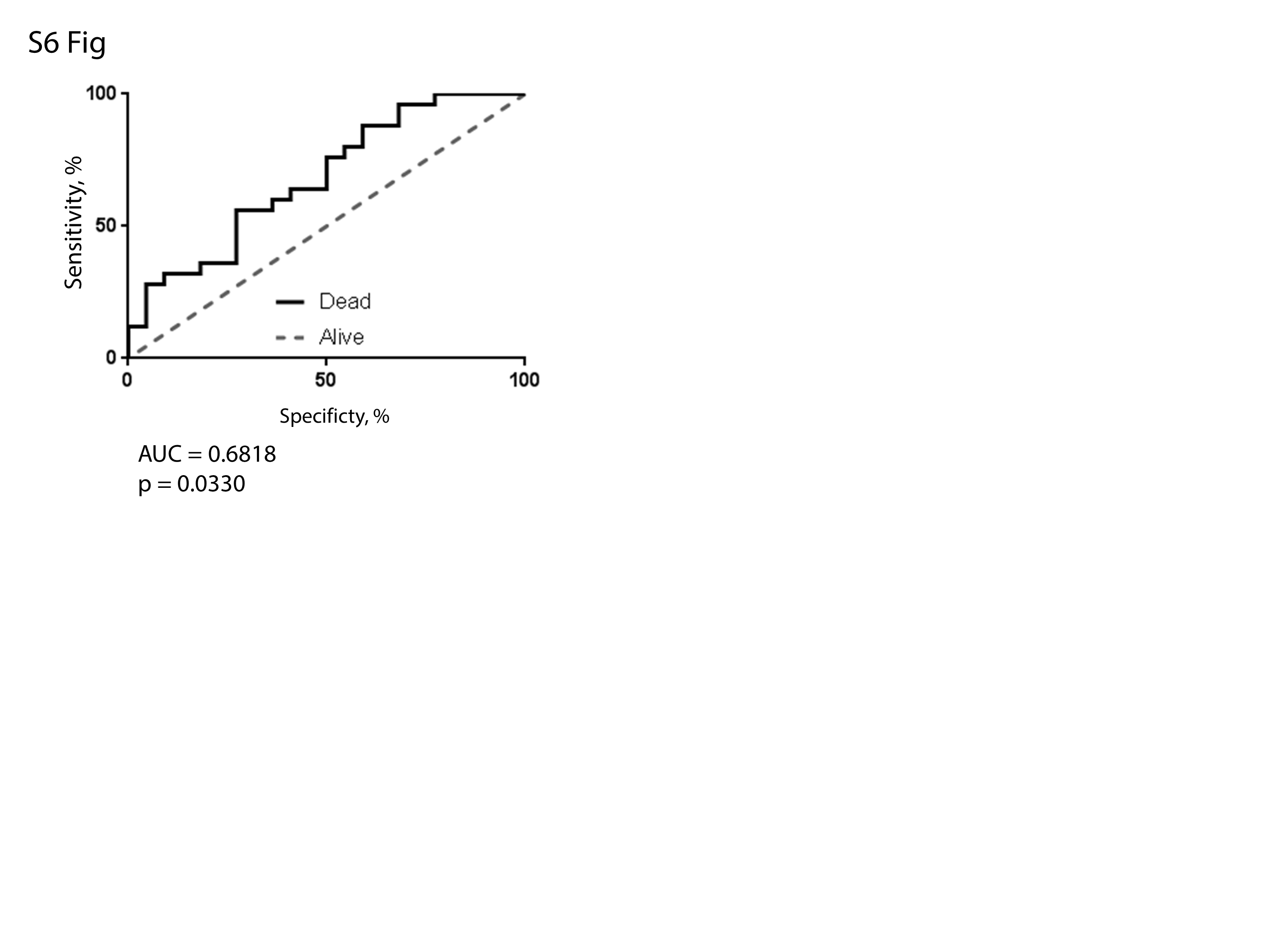

Supplement: S6 Fig — AUC comparison between the levels of sAXL in patients who were alive or dead two years after ipilimumab treatment. (TIF) [file pone.0227187.s006.tif]
