# Supplementary material for: Soluble AXL as a marker of disease progression and survival in melanoma
Source: PLoS One. 2020 Jan 9;15(1):e0227187. doi: 10.1371/journal.pone.0227187 (PMC6952099; doi:10.1371/journal.pone.0227187)
Supplement: S1 Table — Ulceration, gender, age and Breslow depth sorted by high (n = 80) or low (n = 80) sAXL levels. Groups were determined by the median sAXL value. (PDF) [file pone.0227187.s007.pdf]

S1 Table. Patient parameters grouped by median sAXL value

		AXL low (n=80)	AXL high (n=80)	p value
**Ulceration, count**				0.993
	Yes	21	20	
	No	21	21	
	Uknown	38	39	
**Gender, count**				0.655
	Male	51	53	
	Female	29	27	
**Age, median (range)**		65 (31-94)	65 (25-93)	0.813
**Breslow, mm (range)**		2.85 (0.4-9)	4.3 (0.4-25)	0.09
