# Supplementary material for: Soluble AXL as a marker of disease progression and survival in melanoma
Source: PLoS One. 2020 Jan 9;15(1):e0227187. doi: 10.1371/journal.pone.0227187 (PMC6952099; doi:10.1371/journal.pone.0227187)
Supplement: S1 Supplementary Methods — (DOCX) [file pone.0227187.s008.docx]

**Supplementary Methods:**

**mRNA expression from TCGA data:**

The mRNA expression profiles for TIMP1 and AXL gene were analyzed and compared for stage III and stage IV melanoma using Skin Cutaneous Melanoma data (SKCM) from The Cancer Genome Atlas (TCGA Research Network: [https://www.cancer.gov/tcga](https://www.cancer.gov/about-nci/organization/ccg/research/structural-genomics/tcga)). SKCM raw count data (n=470) was downloaded from recount2: analysis-ready RNA-seq gene and exon counts datasets (1) and Bioconductor package recount (2). TCGA clinical data, including the melanoma stage III and stage IV information, was retrieved from NIH, National Cancer Institute, GDC Data portal (3). Prior to analysis, count data was normalized by log2 transformation (log2 + 1) and presented as log2. The mRNA expression profiles in stage III and stage IV were visualized in boxplots (4) and statistically analyzed using Wilcoxon signed-rank test in R (5).

**References:**

1. Collado-Torres L, Nellore A, Kammers K, Ellis SE, Taub MA, Hansen KD, et al. Reproducible RNA-seq analysis using recount2. Nature biotechnology. 2017;35(4):319-21.

2. Collado-Torres L, Nellore A, Jaffe AE. recount workflow: Accessing over 70,000 human RNA-seq samples with Bioconductor. F1000Res. 2017;6:1558.

3. Grossman RL, Heath AP, Ferretti V, Varmus HE, Lowy DR, Kibbe WA, et al. Toward a Shared Vision for Cancer Genomic Data. The New England journal of medicine. 2016;375(12):1109-12.

4. Wickham H. ggplot2: Elegant Graphics for Data Analysis: Springer International Publishing; 2016.

5. R Core Team. R: A language and environment for statistical computing. R Foundation for Statistical Computing, Vienna, Austria Available online at <https://wwwR-projectorg/>. 2018.
