# Supplementary material for: Soluble AXL as a marker of disease progression and survival in melanoma
Source: PLoS One. 2020 Jan 9;15(1):e0227187. doi: 10.1371/journal.pone.0227187 (PMC6952099; doi:10.1371/journal.pone.0227187)

Figure 1

B)

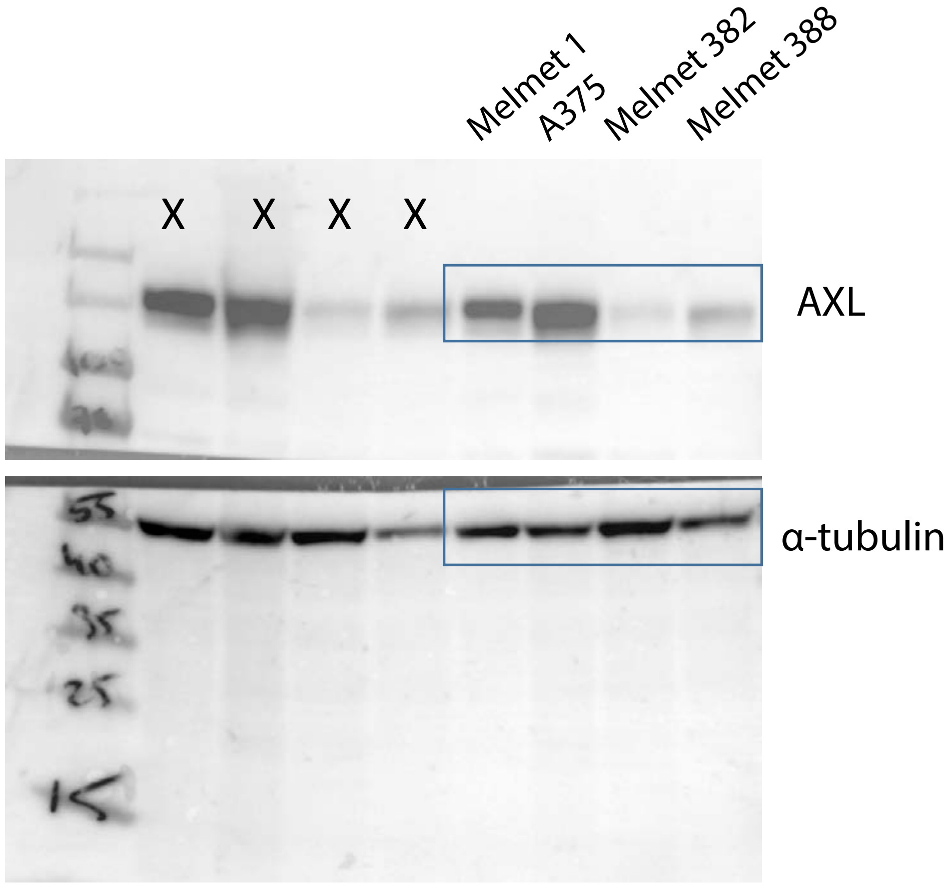
Melmet 1
A375
Melmet 382
Melmet 388
X
X
X
X
AXL
55
40
35
25
15
α-tubulin

Figure 2

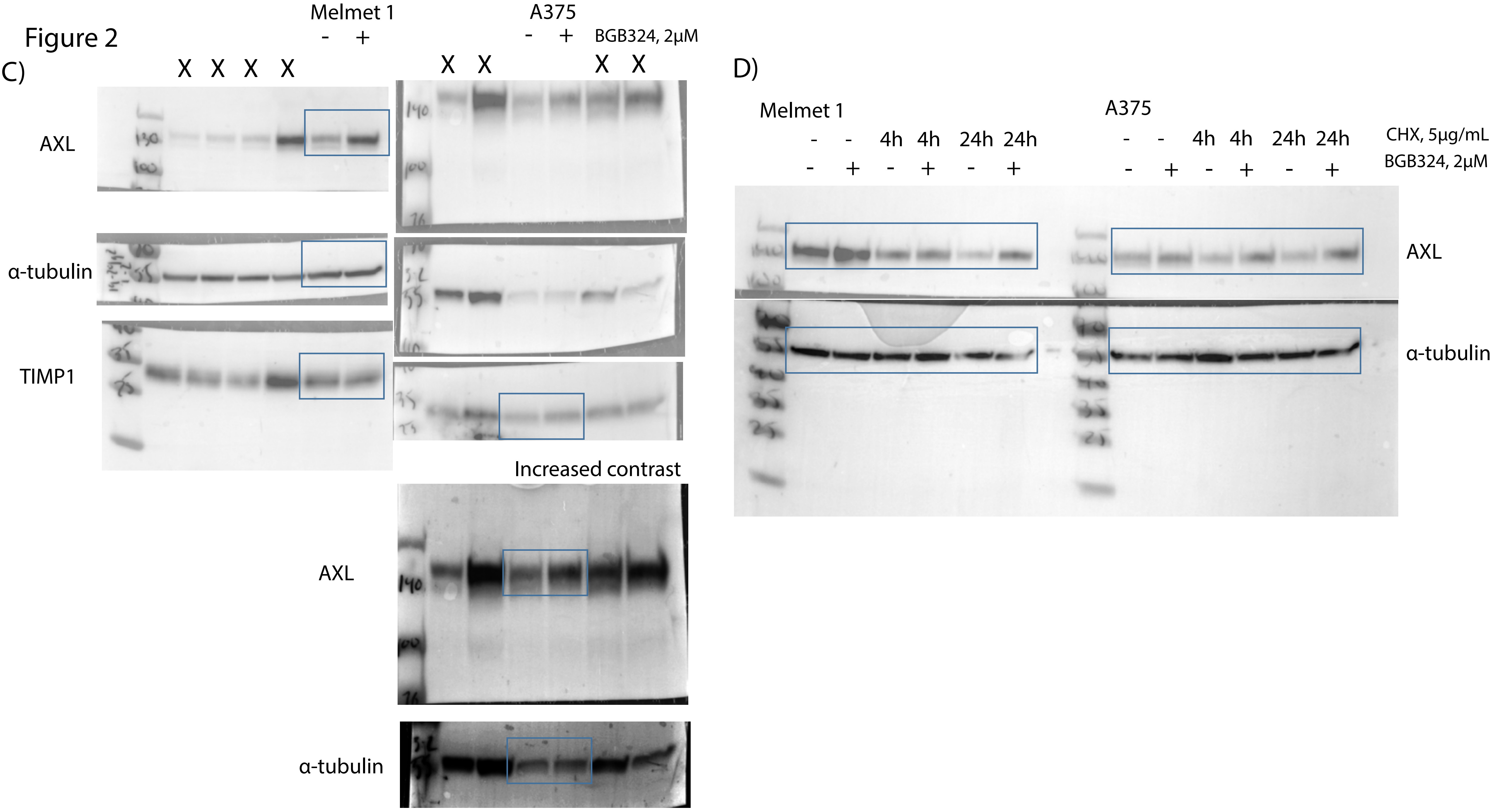

C)
Melmet 1
A375
- + - + BGB324, 2µM
X X X X X X X X
AXL
α-tubulin
TIMP1
Increased contrast
AXL
α-tubulin
D)
Melmet 1
A375
CHX, 5µg/mL
BGB324, 2µM
- - 4h 4h 24h 24h
- + - + - +
AXL
α-tubulin

Figure 3

C)

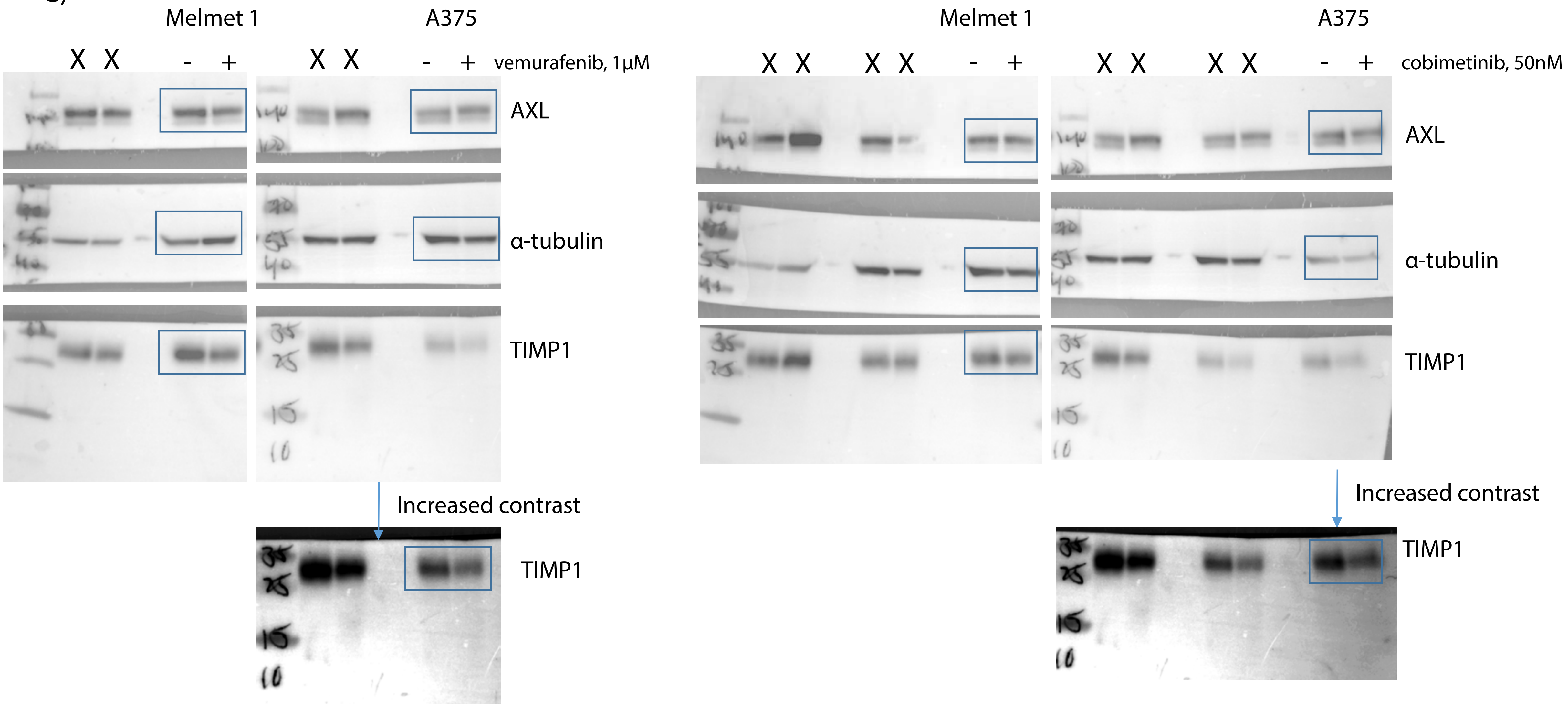
Melmet 1
A375
X X - +
X X - +
vemurafenib, 1µM
AXL
α-tubulin
TIMP1
Increased contrast
TIMP1
Melmet 1
A375
X X X X - +
X X X X - +
cobimetinib, 50nM
AXL
α-tubulin
TIMP1
Increased contrast
TIMP1

## Supplementary Figure 1B

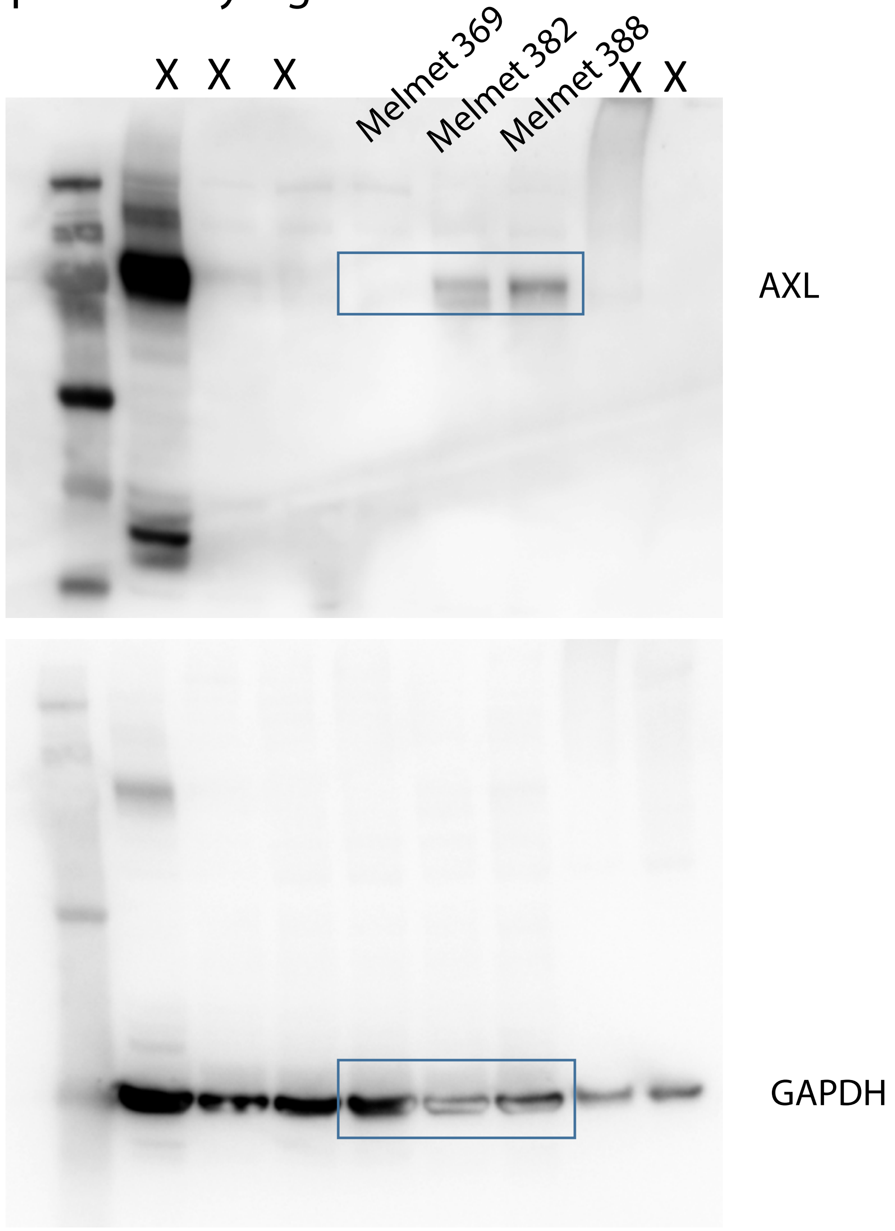


## Supplementary Figure 2B

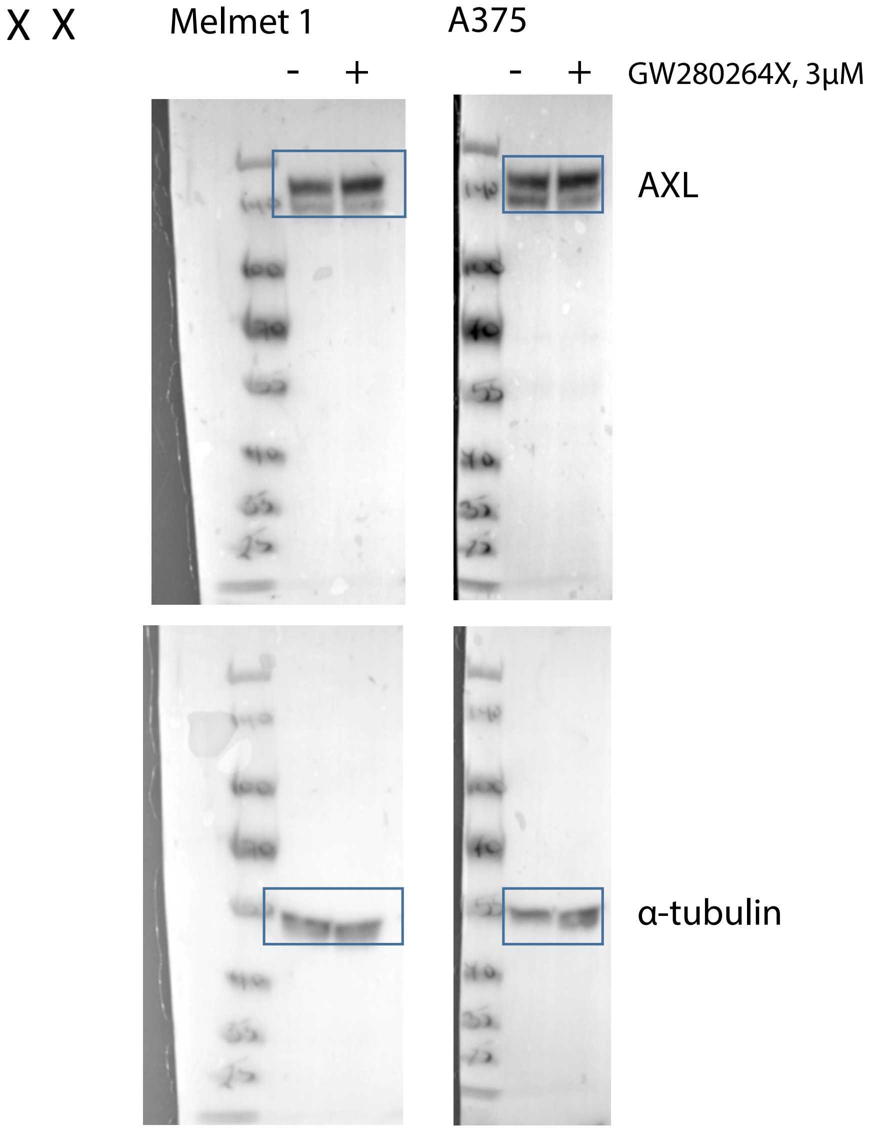

Supplement: S1 Raw Images — Areas used in figures are indicated with blue brackets. (PDF) [file pone.0227187.s009.pdf]
